# Nutritional and physical pathways from feeding difficulty to sarcopenia in Alzheimer’s dementia

**DOI:** 10.3389/fnut.2025.1740327

**Published:** 2026-01-12

**Authors:** Büşra Başar Gökcen, Elif Buse Canpolat, Ferenc Budán, Duygu Ağagündüz, Dávid Szép

**Affiliations:** 1 Department of Nutrition and Dietetics, Fethiye Faculty of Health Sciences, Muğla Sıtkı Koçman University, Muğla, Türkiye; 2 Department of Phytotherapy, Anadolu University, Eskişehir, Türkiye; 3 Medical School, Institute of Physiology, University of Pécs, Pécs, Hungary; 4 Department of Nutrition and Dietetics, Faculty of Health Sciences, Gazi University, Ankara, Türkiye

**Keywords:** Alzheimer’s dementia, feeding patterns, nutritional status, physical performance, sarcopenia

## Abstract

**Background:**

Older adults with Alzheimer’s dementia commonly experience feeding difficulties, malnutrition, reduced physical activity, and heightened sarcopenia risk, yet the interrelationships among these factors are not well understood. This study aimed to examine the associations among nutritional status, physical functioning, and dietary adherence in older adults and to assess how these relationships may differ according to the presence of Alzheimer’s dementia.

**Methods:**

A total of 145 community-dwelling older adults participated, including 60 individuals with Alzheimer’s dementia and 85 without. Nutritional status was assessed using the Nutritional Form for the Elderly (NUFFE), feeding difficulty with the Edinburgh Feeding Evaluation in Dementia (EdFED), dietary adherence with the Mediterranean Diet Adherence Screener (MEDAS), physical fitness with the Physical Fitness and Exercise Activity Levels of Older Adults Scale (PFES), and sarcopenia risk with the SARC-F scale. Group differences were examined using the Mann–Whitney U and chi-square tests. Associations were further analyzed using binary logistic regression and moderated mediation analysis (Model 59).

**Results:**

Individuals with Alzheimer’s dementia exhibited poorer nutritional status (EdFED: 8.0 vs. 2.0; NUFFE: 10.0 vs. 7.0; both *p* < 0.001) and lower adherence to the Mediterranean diet (6.0 vs. 7.0; *p* = 0.050). Physical fitness scores were lower (PFES: 78.0 vs. 73.0; *p* < 0.001), and sarcopenia risk (SARC-*F* ≥ 4) was higher in the dementia group (88.3% vs. 64.7%; *p* = 0.001). Feeding difficulty, nutritional status, and physical function were strongly correlated (*r* = 0.49–0.73; *p* < 0.01). Logistic regression showed that higher EdFED (OR = 1.15; *p* < 0.001) and NUFFE (OR = 1.14; *p* = 0.002) scores were associated with a higher likelihood of dementia, while greater adherence to the Mediterranean diet was associated with a lower likelihood (OR = 0.82; *p* = 0.015). Moderated mediation analyses indicated that only the PFES model showed a significant moderated indirect effect (IMM = −0.251, 95% CI –0.407 to −0.110), whereas the NUFFE and MEDAS models did not (IMM = −0.137 and −0.009, CIs including zero).

**Conclusion:**

Feeding difficulties, malnutrition, reduced physical fitness, and sarcopenia were substantially more common in individuals with Alzheimer’s dementia. Physical fitness emerged as the strongest mediator linking feeding difficulties to sarcopenia, highlighting the need for integrated nutritional and functional interventions in cognitively impaired older adults.

## 1 Introduction

As the global population continues to age at an unprecedented pace, age-related neurodegenerative disorders—particularly Alzheimer’s disease (AD)—have become an increasingly critical public health challenge (1). AD is a progressive neurodegenerative disorder that causes memory loss and widespread cognitive impairments—including deficits in language, reasoning, and behavior—that become severe enough to interfere with daily activities (2). Among these activities, the ability to eat independently is often one of the earliest and most profoundly compromised (3). Feeding-related difficulties are highly prevalent, affecting nearly 50% of individuals within the first 8 years following diagnosis (4).

Cognitive and functional decline in dementia profoundly disrupts the overall process of eating—affecting hunger and thirst regulation, swallowing safety, and the ability to self-feed or recognize food—and this combined deterioration compromises adequate nutritional intake, leading to progressive weight loss and heightened vulnerability to malnutrition (5, 6). These nutrition-related impairments may also accelerate muscle loss and weakness, thereby elevating the risk of sarcopenia in older adults with dementia (7, 8).

Malnutrition and sarcopenia are distinct but interrelated geriatric syndromes that arise from shared mechanisms such as age-related physiological decline, reduced food intake, and chronic inflammation. These overlapping pathways make older adults—particularly those with cognitive impairment—highly susceptible to both conditions. In AD, feeding difficulties, poor nutritional intake, and declining muscle strength frequently converge, accelerating functional decline (9). Recent meta-analytic evidence further shows that approximately one-third of older adults with dementia are malnourished (32.5%), while nearly half (46.8%) are at risk—underscoring the considerable nutritional vulnerability of older adults with dementia (10). Sarcopenia is similarly prevalent: a recent meta-analysis reported that 33.9% of individuals with AD meet diagnostic criteria for sarcopenia, with rates increasing from 31.2% in mild AD to 41.9% in moderate AD—illustrating the substantial overlap between cognitive decline and muscle degeneration (11). Taken together, these findings highlight the need to more clearly delineate the interrelated pathways linking feeding difficulties, malnutrition, and sarcopenia in AD.

Healthy lifestyle choices, particularly diet-related behaviors and regular physical activity, have been shown to help protect against both malnutrition and sarcopenia (12). In this context, the Mediterranean Diet (MD)—rich in anti-oxidant and anti-inflammatory components—is widely recognized as a protective dietary pattern against age-related inflammatory and metabolic disturbances (13). The benefits of this dietary pattern extend to the neurobiological mechanisms implicated in AD. The onset and progression of AD are characterized by the accumulation of amyloid beta (Aβ) plaques and hyperphosphorylated tau neurofibrillary tangles, alongside pervasive neuroinflammation driven by astrocytes, microglia, cytokines, and chemokines (14–18). Evidence suggests that higher adherence to the MD may mitigate these pathological processes by reducing chronic inflammation, decreasing amyloid accumulation, and limiting brain atrophy, thereby potentially slowing the progression of AD in older adults (19, 20).

Physical inactivity is a major modifiable contributor to AD risk—responsible for approximately 12.7% of global cases—and regular activity plays a critical role in maintaining cognitive health (21). A growing body of evidence indicates that structured physical activity may slow AD progression through multiple interrelated biological pathways—including reduced systemic and neuroinflammation, enhanced neuroplasticity, improved metabolic resilience, and upregulated neurotrophic factors—highlighting its value as a modifiable, disease-relevant therapeutic target (22–24).

Given these interconnected mechanisms, understanding how nutrition, physical function, and cognitive decline interact has become a central focus in geriatric research (25). Feeding difficulties—commonly observed in AD—may initiate a cascade of nutritional and functional impairments that ultimately contribute to sarcopenia. Although previous work has examined these factors individually, the extent to which nutritional status, diet adherence, and physical fitness jointly shape the relationship between feeding difficulties and sarcopenia remains insufficiently clarified, particularly in the context of dementia (6, 26–28).

To address this gap, the present study was designed as a cross-sectional observational investigation using data from community-dwelling older adults. The study aims to examine the direct and indirect pathways linking feeding difficulties to sarcopenia through nutritional, dietary, and physical mechanisms, as well as to determine whether these pathways differ according to dementia status. The findings are expected to inform future research aimed at developing more targeted nutritional and physical interventions for older adults.

## 2 Materials and methods

### 2.1 Study design and population

This cross-sectional study was conducted between October and December 2024 among older adults aged 60 years and over who were receiving services from institutions affiliated with the Bursa Provincial Directorate of the Ministry of Family and Social Services. The study included adults aged 60 years or older who were able to undergo face-to-face assessments and had adequate institutional medical records for classification of dementia status. Exclusion criteria comprised acute illness, severe physical disability preventing assessment, and neurological or psychiatric disorders other than Alzheimer’s dementia that could influence cognitive or functional outcomes. Of the total participants, 60 (41.4%) had Alzheimer’s dementia, while 85 (58.6%) did not have dementia.

Participants were assigned to groups based on information provided by the institutional healthcare staff, who reviewed routine clinical evaluations and medical records to identify the presence or absence of Alzheimer’s-type dementia, and therefore the research team did not directly access participants’ full medical records. Those with a documented diagnosis were classified as the Alzheimer’s dementia group, while individuals without any recorded dementia diagnosis constituted the non-dementia group. Because the research team did not include a clinician qualified to conduct an independent diagnostic evaluation or formal dementia staging, severity levels could not be determined.

The study protocol was approved by the Ethics Committee of Muğla Sıtkı Koçman University, Faculty of Medicine and Health Sciences (date: 01/11/2023, protocol-decision no: 230089–132). In addition, research permission was obtained from the Republic of Türkiye Ministry of Family and Social Services, General Directorate of Disabled and Elderly Services, with the official letter dated 28/12/2023 (No: E-84459573-605.01-9586218). Prior to data collection, all participants were informed about the study and provided written informed consent.

### 2.2 Data collection procedures

All questionnaires were administered in a quiet room within the institutional facilities through face-to-face interviews conducted by trained researchers. For participants with Alzheimer’s dementia, the administration process was adapted to accommodate cognitive limitations: researchers read all items aloud in a calm and clear manner, clarified instructions when needed, and provided additional explanations without leading the participant. Participants were encouraged to respond independently whenever possible.

A similar supportive approach was applied to older adults in the non-dementia group when age-related factors such as hearing difficulties or slowed comprehension required clarification or repetition; however, in no instance were responses provided on behalf of the participant. If a participant was unable to answer independently due to cognitive or sensory challenges, caregivers or institutional healthcare personnel offered practical assistance—such as helping with comprehension or repeating questions—without influencing or supplying the participant’s answers. The level of support varied according to everyone’s cognitive and communicative abilities.

No performance-based physical tests were conducted in this study. Physical fitness and sarcopenia risk were assessed using self-report tools such as the PFES and SARC-F scales. When necessary, these instruments were administered in an interviewer-assisted format, whereby the researcher read items and clarified statements, but all responses were provided directly by the participant. This standardized procedure ensured accessibility for all participants while preserving data integrity.

### 2.3 Data collection tools

A structured questionnaire consisting of four main sections was used for data collection. The first section included questions on age, sex, body weight (kg), height (cm), educational status, economic status, subjective physical activity assessment, medication use, water consumption, and general health status. The second section captured nutritional status and eating habits, the third assessed physical activity and functional fitness, and the fourth screened sarcopenia risk.

#### 2.3.1 Edinburgh feeding evaluation in dementia scale

The EdFED Scale was developed by Watson in 1994 to assess feeding difficulties in individuals with dementia. The scale consists of 11 items and can be administered in less than 5 mins. The first 10 items evaluate feeding behaviors during mealtimes. Scoring is as follows: behavior does not occur (“never”): 0 points, occurs 2–3 times per week (“sometimes”): 1 point, occurs more than 4 times per week (“often”): 2 points. The total score from the first 10 items ranges from 0 to 20, with higher scores indicating more severe feeding difficulties. Item 11 identifies the level of assistance required by the patient during meals. The total score can also be used to monitor changes over time. The EdFED includes three sub-factors: indicators of patient difficulty, patient’s need for assistance, and indicators of feeding difficulty (29). The Turkish adaptation demonstrated strong psychometric properties, with a content validity index of 0.95 and confirmation of the original three-factor structure. Reported Cronbach’s *α* values were 0.81, 0.79, and 0.64 for the three subscales (30).

#### 2.3.2 Nutritional form for the elderly

The NUFFE is a 15-item scale with a maximum score of 30 points designed to assess nutritional status in older adults (31). The Turkish adaptation demonstrated acceptable psychometric properties. After removing three items with low factor loadings during exploratory factor analysis, the final 12-item structure loaded onto two subdimensions. Internal consistency was satisfactory (Cronbach’s *α* = 0.74). The scale evaluates two sub-factors: General health and physical limitations and dietary intake and eating assistance. Higher scores indicate a greater likelihood of nutritional problems in older adults (32).

#### 2.3.3 Mediterranean diet adherence screener

Adherence to the Mediterranean dietary pattern was assessed using the 14-item MEDAS, originally developed by Martínez-González et al. within the PREDIMED trial (33). The Turkish adaptation of the MEDAS demonstrated strong psychometric properties. In the validation study, Cronbach’s *α* was 0.83, indicating high internal consistency. The 14-item structure was preserved, and scoring followed the original format, with higher scores reflecting greater adherence to the Mediterranean diet. A total score ≥7 indicates acceptable adherence, whereas scores <7 reflect low adherence (34).

#### 2.3.4 Physical fitness and exercise activity levels of older adults scale

This scale was developed by Mellillo et al. to assess physical fitness levels, perceived motivators and barriers, and exercise frequency among older adults (35). The Turkish adaptation by Yılmaz et al. showed good internal consistency (Cronbach’s *α* = 0.89), with subscale coefficients ranging from 0.78 to 0.88. Following confirmatory factor analysis, the scale was reduced from 41 to 34 items, organized into four subscales—physical fitness (9 items), perceived barriers (13 items), perceived motivators (11 items), and exercise frequency (8 items)—and scored on a 4-point Likert format, with a minimum total score of 34 and a maximum of 136. In this scale, higher scores reflect poorer physical fitness, more perceived barriers, fewer motivators, and more frequent engagement in physical activity, depending on the subscale (36).

#### 2.3.5 Strength, assistance with walking, rise from a chair, climb stairs, and falls test

Developed by Malmstrom et al. in 2016, this questionnaire is used as a rapid screening tool for sarcopenia. The Turkish adaptation was previously validated by Kış, and in the present study the internal consistency of the scale was acceptable, with a Cronbach’s alpha coefficient of 0.60. The scale consists of five components assessing functional changes related to health status in the context of sarcopenia outcomes: strength, assistance with walking, rising from a chair, climbing stairs, and falls. The total score ranges from 0 to 10, with 0–3 points indicating a “healthy” status and scores of 4 or above representing a “symptomatic” status, which also suggests the presence of sarcopenia (37, 38).

#### 2.3.6 Statistical analysis

All analyses were conducted using IBM SPSS Statistics version 30.0 and the PROCESS macro version 4.2 by Andrew F. Hayes (39). A two-tailed *p* < 0.05 was considered statistically significant. Normality of continuous variables was evaluated using Shapiro–Wilk tests and visual assessment via Q–Q plots and histograms, and the results indicated that none of the variables met normality assumptions. Therefore, non-parametric and distribution-free statistical methods were applied throughout.

Descriptive statistics were presented as n (%) for categorical variables and median (interquartile range, IQR) for continuous variables. Between -group comparisons (Alzheimer’s dementia vs. non-dementia) were conducted using the Mann–Whitney U test for continuous variables and Pearson’s χ^2^ test or Fisher’s exact test for categorical variables. To control for Type I error inflation due to multiple parallel group comparisons (sociodemographic, nutritional, and physical function variables), *p*-values were adjusted using the Benjamini–Hochberg False Discovery Rate (FDR) procedure.

Associations between nutritional status, feeding difficulties, physical fitness, and dietary adherence were examined using Spearman’s rank correlation coefficients (*ρ*) due to the non-normal distribution of the variables. To identify independent predictors of Alzheimer’s dementia, sarcopenia, and diet adherence, binary logistic regression analyses were conducted. Each regression model was adjusted for potential confounders, including age, sex, and BMI. Results are reported as odds ratios (OR) with *p*-values.

To examine direct, indirect, and conditional indirect relationships, a moderated mediation analysis (PROCESS Model 59) was conducted. In this model, feeding difficulties (EdFED) were entered as the predictor (the independent variable), sarcopenia (SARC-F) as the outcome (the dependent variable), and nutritional status (NUFFE), diet adherence (MEDAS), and physical fitness (PFES) as mediators. Dementia status (Alzheimer’s vs. non-dementia) was specified as a moderator on all three paths (a, b, and c′). Bootstrapping with 5,000 resamples was used to estimate indirect and conditional indirect effects. Bias-corrected 95% confidence intervals (CIs) were computed, and mediation or moderated mediation effects were considered significant when the CI did not include zero.

Finally, a post-hoc power analysis was conducted using G Power (v3.1) to determine the statistical power of the study given the sample size of 145. The calculation was based on a multiple regression model with six predictors (representing the independent variable, moderator, interaction terms, and covariates). With a medium effect size (f^2^ = 0.15) and an *α* error probability of 0.05, the analysis revealed that the study achieved a statistical power of 0.95 (1–*β* = 0.949), which is well above the conventional threshold of 0.80. Thus, the sample size was deemed sufficient to detect significant effects within the moderated mediation model.

## 3 Results

The demographic and baseline characteristics of the case and control groups are presented in Table 1. The proportion of males and the prevalence of low educational status were significantly lower in the case group compared to the control group (*p* = 0.012 and *p* = 0.006, respectively). Low activity status was more frequent in the case group (*p* = 0.012), and the prevalence of high pharmacological treatment use was significantly higher (*p* < 0.001). No significant difference was observed between the groups in terms of economic status. The mean age was significantly higher in the case group (*p* = 0.049). No significant differences were found between the groups in body mass index, daily number of main meals, or daily number of snacks (*p* > 0.05). Water consumption was significantly lower in the case group (*p* = 0.023).

**Table 1:** Baseline characteristics of Alzheimer’s dementia and non-dementia groups.

Characteristics	Alzheimer’s dementia	non-dementia	*p* values
n	60	85	-
Gender, *male*	27 (45.0%)	56 (65.9%)	**0.012**
Educational status, *low*	42 (70.0%)	40 (47.1%)	**0.006**
Economic status, *low*	24 (40.0%)	39 (45.9%)	0.482
Activity status, *low*	50 (83.3%)	56 (65.9%)	**0.012**
Pharmacological treatment, *high*	51 (85.0%)	45 (52.9%)	**<0.001**
Chewing and swallowing difficulties, *present*	32 (53.3%)	20 (23.5%)	**<0.001**
Age (years)	79.0 (71.0–89.5)	77.0 (70.0–82.0)	**0.049**
Body mass index (kg/m^2^)	25.9 (24.1–28.4)	25.8 (23.0–28.2)	0.682
Water consumption (L)	1.0 (1.0–1.5)	1.5 (1.0–2.0)	**0.023**

Data are presented as frequency (percentage) or median (interquartile range). Benjamini–Hochberg FDR correction was applied for multiple comparisons; after adjustment, gender, educational status, activity level, pharmacological treatment, chewing/swallowing difficulties, and water intake remained statistically significant. Educational status, low: Individuals not included in the formal education system, regardless of literacy. Economic status, low: Individuals reporting that their income is less than their expenses. Activity status, low: Individuals reporting being sedentary or having low physical activity. Pharmacological treatment, high: Use of more than three medications per day. Bold values should indicate statistically significant results (*p* < 0.05).

Nutritional and physical function measures of the case and control groups are presented in Table 2. The case group had significantly higher EdFED global and sub-factor scores compared to the control group (all *p* < 0.001). Similarly, NUFFE global and sub-factor scores were significantly higher in the case group (*p* ≤ 0.019). MEDAS global scores were slightly lower in the case group, but the difference was at the margin of statistical significance (*p* = 0.050), and the proportion of participants with acceptable adherence did not differ significantly between groups (*p* = 0.054).

**Table 2:** Differences in nutritional and physical function indicators by dementia status.

Nutritional measures	Alzheimer’s dementia	non-dementia	*p* values
EdFED, global score	8.0 (3.0–13.75)	2.0 (0.0–7.5)	**<0.001**
Indicators of patient difficulty	1.5 (0.0–5.0)	0.0 (0.0–1.0)	**<0.001**
Patient’s need for assistance	4.0 (3.0–8.0)	2.0 (0.0–4.5)	**<0.001**
Indicators of feeding difficulty	2.0 (1.0–3.0)	1.0 (0.0–2.0)	**<0.001**
NUFFE, global score	10.0 (7.25–14.0)	7.0 (4.0–12.0)	**0.001**
General health and physical limitations	6.0 (4.0–8.0)	5.0 (3.0–6.5)	**0.019**
Dietary intake and eating assistance	5.0 (3.0–7.0)	3.0 (1.0–5.0)	**<0.001**
MEDAS, global score	6.0 (5.0–8.0)	7.0 (5.5–9.0)	0.050
≥7 points %	27 (45.0%)	52 (61.2%)	0.054
Physical function measures			
PFES, global score	78.0 (74.0–86.75)	73.0 (65.0–79.5)	**<0.001**
Exercise frequency	10.0 (9.0–13.0)	12.0 (10.0–16.0)	**0.003**
Physical fitness	24.0 (20.0–27.75)	18.0 (12.0–24.5)	**<0.001**
Perceived barriers	22.0 (21.0–26.0)	23.0 (20.0–27.0)	0.994
Perceived motivators	21.0 (19.0–24.75)	17.0 (13.5–22.0)	**<0.001**
SARC-F, global score	8.0 (5.0–9.0)	5.0 (2.0–8.5)	**<0.001**
≥4 points %	53 (88.3%)	55 (64.7%)	**0.001**
Strength	2.0 (1.0–2.0)	1.0 (0.0–2.0)	**0.002**
Assistance with walking	1.0 (1.0–2.0)	1.0 (0.0–1.0)	**0.003**
Rising from a chair	2.0 (1.0–2.0)	1.0 (0.5–2.0)	**<0.001**
Climbing stairs	2.0 (2.0–2.0)	2.0 (1.0–2.0)	**0.002**
Falls	1.0 (1.0–2.0)	1.0 (0.0–1.0)	**0.007**

Data are presented as frequency (percentage) or median (interquartile range). Benjamini–Hochberg False Discovery Rate (FDR) correction was applied to adjust for multiple comparisons in Table 2; all variables except MEDAS scores remained statistically significant after correction. EdFED, Edinburgh Feeding Evaluation in Dementia. NUFFE, Nutritional Form for the Elderly. MEDAS, Mediterranean Diet Adherence Screener; Cut-off ≥7 indicates acceptable adherence. PFES, Physical Fitness and Exercise Activity Levels of Older Adults Scale. SARC-F, Strength, Assistance with walking, Rise from a chair, Climb stairs, and Falls. Higher SARC-F scores greater sarcopenia risk and cut-off ≥4 indicates high risk for sarcopenia. Bold values should indicate statistically significant results (*p* < 0.05).

Regarding physical function, the case group had a higher PFES global score (*p* < 0.001), lower PFES sub-factor 1 score (*p* = 0.003), and higher PFES sub-factor 2 and 4 scores (p < 0.001 for both). PFES sub-factor 3 scores did not differ significantly between groups (*p* = 0.994). SARC-F global scores and all component scores were significantly higher in the case group (*p* ≤ 0.007). The proportion of participants above the SARC-F cut-off was also significantly higher in the case group (*p* = 0.001).

In Spearman correlations, age was positively associated with EdFED, NUFFE, PFES, and SARC-F, and inversely with BMI (all *p* < 0.05), with no significant association with MEDAS. EdFED correlated strongly with NUFFE (*r* = 0.74) and SARC-F (*r* = 0.70), moderately with PFES, and inversely with MEDAS (*r* = −0.32; all *p* < 0.01). NUFFE showed positive correlations with PFES (*r* = 0.49) and SARC-F (*r* = 0.68) and an inverse correlation with MEDAS (*r* = −0.33; *p* < 0.01). PFES correlated positively with SARC-F (*r* = 0.50) and inversely with MEDAS (*r* = −0.27; *p* < 0.01). BMI was inversely related only to NUFFE (*r* = −0.26; *p* < 0.01; Table 3).

**Table 3:** Spearman’s rank correlation coefficients among nutritional, physical, and dietary measures.

Variables	1	2	3	4	5	6	7
Age (years)	-	−0.276^**^	0.218^**^	0.380^**^	0.182^*^	0.261^**^	−0.081
BMI (kg/m^2^)		-	−0.102	−0.264^**^	0.002	−0.129	−0.016
EdFED			-	0.737^**^	0.522^**^	0.703^**^	−0.324^**^
NUFFE				-	0.489^**^	0.678^**^	−0.329^**^
PFES					-	0.496^**^	−0.269^**^
SARC-F						-	−0.320^**^
MEDAS							-

Values represent Spearman’s correlation coefficients. *p < 0.05 (2-tailed) and **p < 0.01 level (2-tailed). BMI: Body Mass Index. EdFED, Edinburgh Feeding Evaluation in Dementia. NUFFE, Nutritional Form for the Elderly. MEDAS, Mediterranean Diet Adherence Screener. PFES, Physical Fitness and Exercise Activity Levels of Older Adults Scale. SARC-F, Strength, Assistance with walking, Rise from a chair, Climb stairs, and Falls.

In models adjusted for age, sex, and BMI, EdFED was positively associated with Alzheimer’s dementia (OR = 1.153; *p* < 0.001) and sarcopenia (OR = 1.968; *p* < 0.001) and inversely associated with good diet adherence (OR = 0.866; *p* < 0.001). NUFFE showed a similar pattern (dementia: OR = 1.141; *p* = 0.002; sarcopenia: OR = 1.592; *p* < 0.001; diet adherence: OR = 0.854; *p* < 0.001). PFES was positively associated with Alzheimer’s dementia (OR = 1.058; *p* < 0.001) and sarcopenia (OR = 1.077; *p* < 0.001) and inversely with good diet adherence (OR = 0.930; *p* < 0.001). SARC-F was positively related to Alzheimer’s dementia (OR = 1.205; *p* = 0.002) and inversely to good diet adherence (OR = 0.806; *p* < 0.001). MEDAS showed inverse associations with Alzheimer’s dementia (OR = 0.816; *p* = 0.015) and sarcopenia (OR = 0.678; *p* < 0.001). Details are presented in Table 4.

**Table 4:** Logistic regression of nutritional and functional predictors.

Predictors	Alzheimer’s dementia		Sarcopenia		Diet adherence	
	OR	*p*	OR	*p*	OR	*p*
EdFED, *total score*	1.153	**<0.001**	1.968	**<0.001**	0.866	**<0.001**
NUFFE, *total score*	1.141	**0.002**	1.592	**<0.001**	0.854	**<0.001**
SARC, *total score*	1.205	**0.002**	-	**-**	0.806	**<0.001**
MEDAS, *total score*	0.816	**0.015**	0.678	**<0.001**	-	**-**

Values represent odds ratios (OR) and significance levels (p). Separate binary logistic regression models were conducted to examine the association of each predictor with the outcomes. Each model was adjusted for age, sex, and body mass index (BMI). For binary outcomes: 0 = non-dementia, 1 = Alzheimer’s dementia, 0 = no sarcopenia, 1 = sarcopenia present and 0 = low diet adherence, 1 = good diet adherence. BMI, Body Mass Index. EdFED, Edinburgh Feeding Evaluation in Dementia. NUFFE, Nutritional Form for the Elderly. MEDAS, Mediterranean Diet Adherence Screener. PFES, Physical Fitness and Exercise Activity Levels of Older Adults Scale. SARC-F, Strength, Assistance with walking, Rise from a chair, Climb stairs, and Falls. Bold values should indicate statistically significant results (*p* < 0.05).

**Figure 1:**
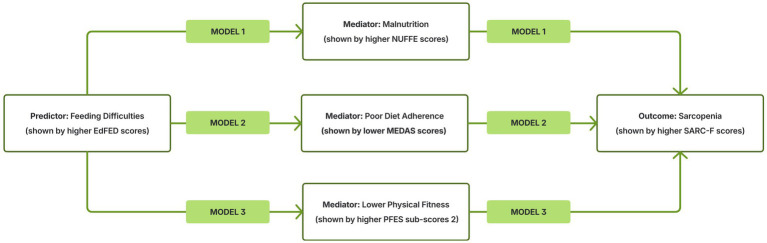
Visual representation of the three moderated mediation models presented in Table 5. This figure illustrates the conceptual structure of the three moderated mediation models tested in the study and reported statistically in Table 5. Model 1: Feeding difficulties → Malnutrition (NUFFE) → Sarcopenia, Model 2: Feeding difficulties → Poor diet adherence (lower MEDAS) → Sarcopenia, Model 3: Feeding difficulties → Lower physical fitness (PFES sub-score 2) → Sarcopenia. In all three models, dementia status (0 = non-dementia, 1 = Alzheimer’s dementia) was examined as a moderator of the a-path (X → M) and/or b-path (M → Y). The corresponding pathway estimates, conditional effects, and the index of moderated mediation (IMM) are provided in Table 5. NUFFE: Nutritional form for the elderly. MEDAS: Mediterranean diet adherence screener. PFES: Physical fitness and exercise activity levels of older adults scale. SARC-F: Strength, assistance with walking, rise from a chair, climb stairs, and falls.

Moderated mediation analyses were conducted to examine whether dementia status moderated the indirect association between feeding difficulties (EdFED) and sarcopenia (SARC-F) through three mediators: malnutrition (NUFFE; Model 1), diet adherence (MEDAS; Model 2), and physical fitness (PFES sub-score 2; Model 3). The detailed statistical results of the three moderated mediation models are presented in Table 5, and their visual representation is provided in Figure 1.

**Table 5:** Mediation pathways between feeding difficulties and sarcopenia by dementia status.

Models/Paths		Effect (B)	SE	LLCI	ULCI	*p*
Model 1	Path a (X → M)	**0.688**	0.076	0.537	0.839	**<0.001**
	Group Interaction	−0.084	0.104	−0.290	0.122	0.420
	Path b (M → Y)	**0.331**	0.072	0.189	0.474	**<0.001**
	Group Interaction	−0.181	0.115	−0.408	0.045	0.116
	Direct effect (c′)	**0.221**	0.071	0.081	0.362	**0.002**
	Group Interaction	−0.022	0.101	−0.221	0.178	0.831
	non-Dementia	**0.221**	0.071	0.081	0.362	**0.002**
	Alzheimer’s Dementia	**0.200**	0.072	0.058	0.341	**0.006**
	Indirect effect (a × b)	**0.173**	0.040	0.099	0.254	**sign.**
	non-Dementia	**0.228**	0.064	0.112	0.361	**sign.**
	Alzheimer’s Dementia	0.091	0.047	−0.001	0.183	ns.
	Index of Moderated Mediation	−0.137	0.080	−0.303	0.011	ns.
Model 2	Path a (X → M)	**−0.119**	0.048	−0.214	−0.024	**0.015**
	Group Interaction	0.006	0.066	−0.124	0.136	0.932
	Path b (M → Y)	−0.202	0.122	−0.442	0.039	0.100
	Group Interaction	0.069	0.195	−0.316	0.453	0.725
	Direct effect (c′)	**0.425**	0.056	0.314	0.537	**<0.001**
	Group Interaction	−0.150	0.078	−0.303	0.003	0.055
	non-Dementia	**0.425**	0.056	0.314	0.537	**<0.001**
	Alzheimer’s Dementia	**0.275**	0.053	0.170	0.381	**<0.001**
	Indirect effect (a × b)	0.023	0.015	−0.004	0.054	ns.
	non-Dementia	0.024	0.020	−0.011	0.070	ns.
	Alzheimer’s Dementia	0.015	0.021	−0.018	0.067	ns.
	Index of Moderated Mediation	−0.009	0.029	−0.065	0.054	ns.
Model 3	Path a (X → M)	**0.858**	0.073	0.713	1.003	**<0.001**
	Group Interaction	**−0.533**	0.152	−0.834	−0.232	**<0.001**
	non-Dementia	**1.099**	0.116	0.867	1.330	**<0.001**
	Alzheimer’s Dementia	**0.566**	0.097	0.372	0.759	**<0.001**
	Path b (M → Y)	**0.295**	0.031	0.234	0.357	**<0.001**
	Group Interaction	−0.124	0.068	−0.259	0.011	0.072
	non-Dementia	**0.340**	0.043	0.254	0.426	**<0.001**
	Alzheimer’s Dementia	**0.216**	0.050	0.116	0.317	**<0.001**
	Direct effect (c′)	**0.122**	0.038	0.046	0.198	**0.002**
	Group Interaction	0.092	0.081	−0.067	0.252	0.253
	non-Dementia	0.076	0.066	−0.055	0.207	0.254
	Alzheimer’s Dementia	**0.168**	0.047	0.075	0.261	**<0.001**
	Indirect effect (a × b)	**0.253**	0.035	0.188	0.327	**sign.**
	non-Dementia	**0.374**	0.061	0.272	0.512	**sign.**
	Alzheimer’s Dementia	**0.122**	0.044	0.046	0.217	**sign.**
	Index of Moderated Mediation	**−0.251**	0.075	−0.407	−0.110	**sign.**

Values represent unstandardized regression coefficients (B) with standard errors (SE) and 95% confidence intervals (LLCI–ULCI). Path a refers to the effect of feeding difficulties (EdFED) on the mediator (M); Path b refers to the effect of the mediator on sarcopenia (SARC-F); and the direct effect (c′) represents the association between EdFED and SARC-F after controlling for the mediator. “Group Interaction” denotes the moderation effect of dementia status (0 = non-Dementia, 1 = Alzheimer’s Dementia) on each path. Conditional effects (Path a, Path b, Path c′, and indirect effects) are presented separately for both dementia groups. Indirect effects were estimated using 5,000 bootstrap samples with 95% percentile confidence intervals. A significant indirect effect is indicated when the bootstrap confidence interval does not include zero. The Index of Moderated Mediation (IMM) quantifies whether the indirect effect differs between dementia groups; IMM confidence intervals including zero indicate non-significant moderated mediation. NUFFE, Nutritional Form for the Elderly. MEDAS, Mediterranean Diet Adherence Screener. PFES, Physical Fitness and Exercise Activity Levels of Older Adults Scale. SARC-F, Strength, Assistance with walking, Rise from a chair, Climb stairs, and Falls. Bold values should indicate statistically significant results (*p* < 0.05).

Feeding difficulties significantly predicted NUFFE (B = 0.688, *p* < 0.001), with no moderation by dementia status (*p* = 0.420). NUFFE significantly predicted SARC-F (B = 0.331, *p* < 0.001), and the NUFFE × Group interaction was non-significant (*p* = 0.116). The direct effect of EdFED on SARC-F was significant in both groups. The indirect effect through NUFFE was significant in the non-dementia group (B = 0.228, BootLLCI = 0.112, BootULCI = 0.361) but not in the Alzheimer’s group. The Index of Moderated Mediation (IMM) was non-significant (IMM = −0.137, BootLLCI = −0.303, BootULCI = 0.011).

Feeding difficulties predicted lower MEDAS scores (B = −0.119, *p* = 0.015), with no moderation by dementia status (*p* = 0.932). Neither MEDAS nor its interaction with group significantly predicted SARC-F. Indirect effects through MEDAS were non-significant in both groups. The IMM was non-significant (IMM = −0.009, BootLLCI = −0.065, BootULCI = 0.054).

Feeding difficulties strongly predicted lower physical fitness (B = 0.858, *p* < 0.001), and this association was significantly moderated by dementia status (interaction B = −0.533, *p* < 0.001), with a larger effect in the non-dementia group. Physical fitness significantly predicted SARC-F (B = 0.295, *p* < 0.001), with a marginal interaction (*p* = 0.072). Indirect effects were significant in both groups, but substantially stronger in the non-dementia group (non-Dementia: B = 0.374; Alzheimer’s: B = 0.122). The IMM was significant (IMM = −0.251, BootLLCI = −0.407, BootULCI = −0.110), indicating that the magnitude of mediation differed by dementia status.

## 4 Discussion

This study demonstrated that individuals with Alzheimer’s dementia showed higher levels of feeding difficulties, poorer nutritional status, lower physical fitness, and a greater risk of sarcopenia compared to those without dementia. Logistic regression analyses revealed that greater feeding difficulties, poorer nutritional status, and higher sarcopenia scores were significantly associated with both dementia and sarcopenia. In addition, lower adherence to the Mediterranean diet was found to be associated with higher odds of both dementia and sarcopenia. Mediation analyses indicated that poorer nutritional status explained the relationship between feeding difficulties and sarcopenia only in the non-dementia group, whereas physical fitness mediated this association in both groups, with a stronger effect observed among individuals without dementia.

Age related physiological changes in the swallowing mechanism are described as presbyphagia. Reduced salivary flow, problems related to teeth and dentures, decreased sensory sensitivity in the oral and pharyngeal structures, loss of muscle strength and function, and a decline in the adaptive capacity of the brain constitute the main components of this process. Together, these factors make the safe and efficient transfer of food from the mouth to the stomach more difficult and increase vulnerability to dysphagia (40, 41). When the neuropathological changes characteristic of AD are added to this age-related physiological vulnerability, swallowing impairments tend to become even more pronounced. In AD, dysphagia is thought to arise from functional alterations in the cortical swallowing network as well as autonomic dysfunction affecting the oral and pharyngeal phases of swallowing. As dementia progresses, this compounded vulnerability may intensify swallowing difficulties (42, 43). In our study, individuals with AD exhibited significantly higher EdFED scores, and logistic regression analyses indicated that these scores predicted dementia status. This pattern suggests that feeding difficulties may be more common and possibly more severe in this population. Furthermore, our findings demonstrated that chewing and swallowing difficulties were reported considerably more often by individuals with AD than by those without dementia (53.3% vs. 23.5%).

It is well established that more than half of individuals living with dementia report difficulties related to eating, drinking and swallowing (44, 45). Although such problems may appear even in the early stages of the disease, the prevalence of dysphagia increases markedly as dementia progresses, reaching between 84 and 93% in moderate to severe stages (46). This progression can progressively impair essential physical abilities required for eating, including lip and tongue movements, retention of food in the mouth and actions such as gargling, as well as subjective aspects of eating, such as appetite, food preferences and eating habits. Consequently, both solid and liquid intake may become restricted, thereby increasing the risk of body weight loss, malnutrition and dehydration (46, 47).

In line with this pattern, our study showed that individuals with Alzheimer’s dementia had significantly lower daily water consumption and markedly higher NUFFE scores. Moreover, the logistic regression results demonstrated that this nutritional measure predicted dementia status, suggesting that both malnutrition and dehydration risks may be greater in this group. Meta-analysis findings indicate that malnutrition is common among individuals with dementia living in long-term care facilities, with a pooled prevalence of approximately 57% (6). Globally, it has been reported that about one-third (32.5%) of older adults with dementia are diagnosed with malnutrition, and nearly half (46.8%) are at risk of malnutrition (10). Furthermore, a study examining the relationship between AD and various nutritional assessment tools found that lower scores on the Mini Nutritional Assessment (MNA) and the Geriatric Nutritional Risk Index (GNRI) were strongly associated with the onset and progression of the disease (48). Similarly, another study showed that 64.8% of individuals with Alzheimer’s disease had malnutrition or were at risk of malnutrition, and that this condition became more pronounced as the stage of the disease advanced (49).

In addition to malnutrition, other conditions that commonly co-occur with swallowing difficulties and are frequently observed in individuals with Alzheimer’s disease include frailty and sarcopenia. Frailty, which involves low muscle strength, slow physical performance, fatigue, reduced physical activity and unintentional weight loss, is closely related to sarcopenia, a condition marked by reduced muscle strength and muscle mass (42, 50–52). In this study, individuals with Alzheimer’s dementia had significantly higher SARC-F total scores, and a greater proportion of participants fell into the high-risk category (≥4 points). Furthermore, logistic regression analysis showed that the SARC-F total score was significantly associated with Alzheimer’s dementia, suggesting that physical frailty and impaired muscle function may be related to the condition. A recent meta-analysis demonstrated that sarcopenia was significantly associated with Alzheimer’s disease, showing a nearly threefold higher odds (OR = 2.97, 95% CI 2.15–4.08) (53). Similarly, another meta-analysis reported that approximately one-third of patients with Alzheimer’s disease experienced sarcopenia (33.9%), and the odds of sarcopenia were about 2.7 times higher compared to cognitively healthy individuals (OR = 2.67, 95% CI 1.56–4.55) (11). Recent studies have suggested a possible association between sarcopenia and Alzheimer’s disease, proposing that these two conditions may share common pathological mechanisms such as chronic inflammation, oxidative stress, and metabolic dysfunction. Moreover, these conditions may mutually exacerbate one another, leading to a vicious cycle of progressive deterioration in both physical and cognitive health (11). Given this complex interplay, recent research has increasingly examined modifiable lifestyle factors, including anti-inflammatory and antioxidant dietary patterns, to better understand their potential associations with cognitive aging (54, 55).

Meta-analyses, systematic reviews, and large cohort studies suggest that higher adherence to the Mediterranean diet may be associated with approximately 11–30% lower risk levels for age-related cognitive disorders, including cognitive decline, dementia, and Alzheimer’s disease (56–59). A cross-sectional study reported significantly lower MEDAS scores among individuals with AD, and another study suggested that higher adherence to the dietary pattern may be associated with better cognitive performance (60, 61). Furthermore, a meta-analysis examining the effects of the Mediterranean diet on dementia-related brain magnetic resonance imaging markers reported a significant inverse association between adherence to the Mediterranean diet and white matter hyperintensity (62). Although the difference in Mediterranean diet adherence scores between the groups did not reach statistical significance (*p* = 0.050), a tendency toward higher adherence was observed among individuals without dementia. Logistic regression analysis further indicated that higher MEDAS scores were associated with lower odds of dementia. In line with this finding, the proportion of participants with high adherence (MEDAS ≥ 7) was greater in the non-dementia group than in the dementia group (61.2% vs. 45.0%).

Our model results indicate that the relationship between feeding difficulties and sarcopenia operates through different mediating mechanisms in the two groups. The first model showed that feeding difficulties were positively associated with both malnutrition and sarcopenia, and that malnutrition was also linked to sarcopenia. However, the indirect pathway from feeding difficulties to sarcopenia through malnutrition reached significance only in the non-dementia group. This suggests that, in individuals with Alzheimer’s disease, the association between feeding difficulties and sarcopenia may not be fully explained by nutritional status and may instead reflect a more complex pattern influenced by dementia specific biological processes. This interpretation is consistent with studies reporting that skeletal muscle mass (SMMI), physical performance (TUG), and functional capacity indicators such as Activities of Daily Living (ADL) and Instrumental Activities of Daily Living (IADL) do not differ significantly across nutritional status categories in Alzheimer populations. Such findings imply that sarcopenic processes in this group may progress through biological or neurological mechanisms that are less directly dependent on nutrition (63). Similarly, fear of falling, which is known to be associated with physical activity limitations, functional decline, and cognitive impairment, has been reported to correlate with sarcopenia in individuals with dementia, whereas no significant association has been observed with malnutrition. This pattern suggests that the influence of nutritional status on physical and functional outcomes may be relatively limited in the Alzheimer group (27).

According to the second model, adherence to the Mediterranean diet did not exhibit a significant mediating role in the association between feeding difficulties and sarcopenia. Indeed, a recent systematic review reported that although adherence to the Mediterranean diet is positively associated with muscle mass and muscle function, its effects on muscle strength are inconsistent and there is no clear evidence that the diet prevents or improves sarcopenia (64). Similarly, another review noted that while some studies suggest potential beneficial effects of the Mediterranean diet on sarcopenia and frailty, the overall evidence remains inconclusive. Nonetheless, this review also highlighted that when combined with physical activity, the Mediterranean diet may exert favorable effects on indicators related to sarcopenia (65).

In the third model, the association between feeding difficulties and physical fitness, as well as the mediating role of physical fitness in the relationship between feeding difficulties and sarcopenia, was observed to be stronger in the non-dementia group, despite operating in the same direction across both groups. This finding is further supported by the significant Index of Moderated Mediation (IMM), indicating that the strength of the mediation effect varies according to dementia status. These results suggest that physical fitness may represent a more central component of sarcopenic processes in individuals without dementia. Moreover, the observation that the direct association between feeding difficulties and sarcopenia reached significance only in the Alzheimer group supports the likelihood of full mediation by physical fitness in non-dementia individuals and partial mediation in those with Alzheimer’s disease.

The physiological framework underlying this difference is also noteworthy. The age-related decline in skeletal muscle mass results from the interplay of molecular, neurological, cellular, and metabolic mechanisms and is considered part of the complex process known as primary sarcopenia. One of the main determinants of primary sarcopenia is physical inactivity, which may help explain why physical fitness demonstrated a stronger mediating effect in individuals without dementia (66). In contrast, the development of sarcopenia in Alzheimer’s disease is known to be influenced not only by physical inactivity but also by disease specific neurological degeneration and metabolic disturbances. The strong association observed between Alzheimer’s disease and sarcopenia suggests that sarcopenia may not merely reflect age related decline but could also be linked to the underlying pathophysiology of Alzheimer’s disease (11). Considering this distinction, the stronger mediating role of physical fitness observed in the non-dementia group aligns with characteristics of primary sarcopenia, whereas the weaker mediation observed in individuals with Alzheimer’s disease resembles secondary sarcopenia, which has been associated with processes such as inflammation, neurological damage, hormonal alterations, or metabolic dysfunction (67). Supporting this, a study reported that sarcopenia remained significantly associated with global cognitive performance even after adjusting for obesity, physical activity levels, and comorbidities, indicating that sarcopenia may serve as an independent risk factor for cognitive decline (68). Overall, the significant IMM indicates that the strength of the indirect effect through physical fitness differs by dementia status. In other words, the mediating role of physical fitness is substantially attenuated in individuals with Alzheimer’s dementia, suggesting that disease-related factors may limit the extent to which physical fitness explains the association between feeding difficulties and sarcopenia.

### 4.1 Study limitations

This study has several limitations that should be considered when interpreting the findings. First, the cross-sectional design prevents causal inferences regarding the observed associations between feeding difficulties, nutritional status, physical function, and sarcopenia risk. Second, although the sample size was adequate for statistical analyses, participants were recruited from institutions within a single geographic region, which may limit generalizability to broader older adult populations. Third, nutritional and physical function measures relied on self-reported data for certain parameters (e.g., dietary adherence, physical activity frequency), which may be subject to recall bias. These measures may be prone to recall or response bias, particularly among individuals with Alzheimer’s dementia.

Importantly, cognitive impairment severity within the Alzheimer’s dementia group was not assessed or stratified. The diagnosis was obtained solely from routine clinical evaluations and medical records reviewed by institutional healthcare staff, as the research team did not independently access full medical files or include a clinician qualified to verify diagnoses or conduct formal staging. As a result, dementia severity levels (e.g., mild, moderate, severe) could not be determined, which may affect the interpretation of findings and the accuracy of self-reported measures among participants with dementia. Fourth, baseline differences between groups—particularly in activity status and chewing/swallowing difficulties—may have introduced residual confounding despite statistical adjustments. These imbalances should be taken into consideration when interpreting group comparisons. Lastly, biochemical markers of nutritional status and muscle function were not included, restricting the physiological depth of the analyses.

## 5 Conclusion

This study demonstrates that feeding difficulties, poor nutritional status, reduced physical fitness, and elevated sarcopenia risk are substantially more prevalent among individuals with Alzheimer’s dementia compared with those without dementia. Both feeding difficulties and inadequate nutritional status emerged as strong predictors of dementia and sarcopenia, and lower adherence to the Mediterranean diet was associated with increased risk of these conditions. Moderated mediation analyses revealed that physical fitness consistently mediated the relationship between feeding difficulties and sarcopenia across groups, whereas nutritional status served as a mediator only in individuals without dementia. These findings suggest that early attention to nutrition, physical fitness and swallowing related difficulties may help reduce vulnerability to sarcopenia in older adults, particularly in those with cognitive impairment.

## Data Availability

The raw data supporting the conclusions of this article will be made available by the authors, without undue reservation.
